# Sex Differences in HIV Infection: Mystique Versus Machismo

**DOI:** 10.20411/pai.v3i1.238

**Published:** 2018-07-13

**Authors:** Eileen P. Scully

**Affiliations:** 1 Department of Medicine, Division of Infectious Diseases, Johns Hopkins University School of Medicine

**Keywords:** HIV, gender, sexually transmitted diseases, immune response

## Abstract

Biological sex is a determinant of both susceptibility to and pathogenesis of multiple infections, including HIV. These differences have effects on the spectrum of HIV disease from acquisition to eradication, with diverse mechanisms including distinct chromosomal complements, variation in microbiota composition, hormonal effects on transcriptional profiles, and expression of different immunoregulatory elements. With a comparative biology approach, these sex differences can be used to highlight protective and detrimental immune activation pathways, to identify strategies for effective prevention, treatment, and curative interventions.

## INTRODUCTION

Social, behavioral, and biological differences between men and women have a clear influence on the natural history of disease and the response to therapeutic interventions. The fact that men and women are different is simultaneously treated as manifest and inconsequential in many scientific studies; traditionally, medical science has approached sex as a source of variation requiring controlled analysis, but there has been limited investigation into the mechanisms of these differences. Within the spectrum of HIV disease, sex differences have a greater significance, and these differences are critical to understand for the design of clinical interventions and trials. Furthermore, the distinctions between men and women offer a unique point of leverage for defining disease pathways that can be modulated for the goals of vaccine-induced protection, attenuated pathogenesis, or cure. This review outlines the data's delineating sex-based differences in acquisition, disease progression, and potential for cure. Potential mechanisms for these differences are highlighted, linking HIV to other infections and vaccine responses and emphasizing the areas in need of further research. For each of these sections, the known differences are placed in the context of their implications for current clinical challenges for HIV prevention, management of the complications of disease, and efforts at eradication.

## ACQUISITION

The emergence of HIV in men who have sex with men (MSM) in the United States [1] is in contrast to the modern, mature HIV epidemic where a significant burden of infection worldwide is borne by women; 59% of infections in Sub-Saharan Africa and 51% of total infections worldwide are in women and girls [2]. Although the epidemic is generalized, important sex distinctions with respect to disease acquisition remain. Behavioral and socioeconomic factors drive a substantial proportion of the difference in acquisition between men and women. This was highlighted by early studies suggesting lower efficacy of pharmacologic pre-exposure prophylaxis in women, a difference ultimately attributed to very low adherence in some studies of women [3–5]. However, biological determinants of host susceptibility are modulated by sex, substantially contributing to risk differentials [6]. Sex-based variation offers critical insights into pathways that may be relevant to prevention efforts with either pharmacologic strategies or vaccination.

### Anatomy, microbiome, and sexually transmitted diseases

Male to female sexual transmission is more efficient; penile-vaginal sex carries an approximately 2-fold higher risk of HIV acquisition for the receptive partner versus the insertive partner in a serodiscordant encounter [7]. The portal of entry for sexual transmission is a primary difference, with significant differences in epithelial barrier function and immune cell complement between the penile and rectal mucosal surfaces and the female genital tract. In the female genital tract enhanced susceptibility is associated with local inflammation [8, 9]; data suggest that specific patterns of cytokines and chemokines associate with a local influx of cells susceptible to HIV infection and with changes in the expression of factors promoting mucosal barrier maintenance [10]. This may facilitate transmission of less fit viruses [11] and increase the likelihood of infection. Sexually transmitted infections (STIs) are one driver of inflammation in the genital tract [12, 13] and have been linked to enhanced transmission of HIV [14–16] in both men and women. Of note, women have an enhanced risk of HIV-infection associated with laboratory diagnosed STIs, even in the absence of clinically symptomatic infection[17].

Growing evidence suggests that another significant contributor to inflammation in the female genital tract is the local microbiome [18–20]. In addition to effects on inflammation and STI coin-fection, microbiome composition has been linked to alterations in genital tract wound healing [21], another pathway of susceptibility. Recent data also directly implicate microbial metabolism with altered drug levels and reduced efficacy seen with specific microbiome patterns [22]. Taken together, data indicate that the balance of local bacteria, including commensals and pathogens, and resulting inflammation and local tissue effects can profoundly impact the risk of HIV acquisition; these factors have clear anatomical differences in men and women with implications for prevention strategies. In the specific case of tenofovir, the local metabolism by microbial communities described above will reduce efficacy in women [22], whereas data from studies in men suggest that tenofovir gel exposure is associated with downregulation of RNA coding for inflammatory regulators in rectal biopsies, potentially increasing protective effects [23].

### Sex Hormones

The risk in the female genital tract is then further modulated by the exposure to sex hormones. Animal studies have previously identified a link between SIV acquisition and high progesterone levels either due to menstrual cycle variation [24–26] or to exogenous administration of proges-tins, modeling contraceptives [27, 28]. In humans, the data are difficult to parse. Progestin-related thinning of vaginal mucosal surfaces in non-human primates (NHPs) is well described, [27–29] but the data in women have shown little or no change in vaginal epithelial thickness in association with depot medroxyprogesterone (DMPA) use [30–34]. Despite this clear difference from the primate model, the preponderance of data does support an increase in HIV acquisition risk in association with DMPA use, with meta-analysis estimates of a hazard ratio in the range of 1.4; no association with other contraceptives has been consistently observed [35–37]. Recent data from a prospective cohort have suggested that there are higher numbers of HIV-susceptible target cells in the cervix in the setting of higher progesterone (based on menstrual cycle or DMPA administration) [38], indicating one potential mechanism for increased acquisition. The effects of hormonal contraception also intersect with risks associated with local microbiota as recent data have also shown that vaginal inflammatory profiles are modulated by both hormonal contraception and local microbiota and concurrent sexually transmitted infections [39].

### Sex differences in the immune response

Finally, the efficacy of vaccination to block acquisition may also vary between the sexes. Genetic and hormonal factors combine to produce sex-specific variations in the immune responses to vaccines as measured by both efficacy and adverse effects [40]. Examples include the increased risk of women of childbearing age to the rare complication of fatal visceroptropic disease following receipt of the yellow fever vaccine [41, 42] and the HSV glycoprotein vaccine which had protective efficacy in women but not in men [43]. Sex differences in HIV vaccine responses are still a subject of exploration. In the only vaccine trial with a protective effect (RV144), the efficacy was 25.8% in men (n = 4875) and 38.6% in women (n = 3085) although this difference was not statistically significant [44]. Potential mechanisms for differences in vaccine efficacy and safety include immune subset variations and differences in pathogen sensing and inflammatory pathway activation. A secondary analysis of RV144 reported sex differences in circulating levels of myeloid and plasmacytoid dendritic cells (pDCs) and a subset of natural killer (NK) cells although these values were not specifically related to protective efficacy [45]. Transcriptional signatures induced by the yellow fever vaccine identified gene programs critical to vaccine-induced protection [46] and a sex-stratified analysis of this data identified distinct gene pathways activated in men versus women [47]. These variations suggest that adjuvant strategies, antigen dose, or site of delivery could all potentially be optimized for more efficacious vaccines. Given the challenges in designing an effective vaccine for prevention of HIV, exploiting immunologic differences between men and women may illuminate pathways critical to generating protective immunity.

From a genetic perspective, the sex chromosomes carry immunoregulatory genes. The X chromosome carries *TLR7* and *TLR8* (both encoding pathogen sensors, the former tightly linked to type 1 interferon production) and *FOXP3* (a transcription factor critical for regulatory T cell development) and a high density of regulatory micro RNA sequences [48–50]. Specific Y chromo-some polymorphisms have also been associated with infection susceptibility suggesting immune activity [51, 52]. While there is not a strict gene dosage effect (due to X inactivation), having two X chromosomes even in the setting of phenotypic male sex (ie Klinefelter's syndrome, XXY) has immunologic consequences; it is associated with increased prevalence of the female predominant autoimmune disease systemic lupus erythematosus (SLE) [53]. Recent work has demonstrated the variability of X chromosome inactivation and consequences for disease pathogenesis [54–56]; biallelic expression of *TLR7* has recently been demonstrated and linked to the development of auto-immune disease [57]. Taken together, the evidence suggests the sex chromosome complement has important consequences for immune function and may drive different inflammatory pathways.

Superimposed on the genetic differences are the effects of sex hormones, including estrogen, progesterone, and androgens, all modulators of immune function. With the caveat that *in vitro* modeling of hormonal milieu is difficult, there is evidence for immune suppressive effects of progesterone on multiple cell types and for concentration-dependent proinflammatory effects of estrogen on specific cells [51]. Furthermore, estrogen, through upregulation of interferon regulatory factor 5 (IRF5) has been reported to enhance type 1 interferon production in response to TLR7 stimulation [58]. Importantly, multiple diverse genes have estrogen regulatory elements in their promoter regions, suggesting that they may be directly controlled by sex hormone exposure. The effects of sex hormones may directly modulate both vaccine efficacy and the natural development of immunity in HIV infection.

### Implications

Sex differences in acquisition offer insights critical to the development of effective prevention efforts. In addition to important behavioral determinants that differ for men and women, sex confers distinct biological risks. This includes factors that enhance transmission through changes in barrier maintenance, target cell availability, local inflammation, and prophylactic drug concentrations as well as variations in vaccine response. There is an urgent need to fully define the role of exogenous hormones given to women as contraception; the critical role of effective contraception is clear, but identifying the methods most compatible with prevention of HIV transmission should share priority. Sex differences in vaccine responses should also be clearly defined as they may point to optimal adjuvant strategies that will be effective in both sexes.

## IMMUNE RESPONSE AND PATHOGENESIS

### Viral load

Once HIV infection has been established sex differences persist and in some cases amplify. Early in the HIV epidemic, work that first demonstrated the link between measures of viral activity and disease progression also highlighted a difference in baseline viral loads between men and women [59]. Several subsequent studies identified similar differences in HIV viral load [60–65], although other studies did not recapitulate this finding [66, 67]. Taken together, the preponderance of the data indicates that women have lower levels of HIV RNA, although there is some convergence in this measure over time and with disease progression [68, 69]. This subtle difference in viral load raised important clinical questions: rates of disease progression did not differ between men and women, suggesting that the viral load thresholds did not adequately identify women at risk for progressive disease. Antiretroviral therapy guidelines initially incorporated viral load measures, leading to significant differences in treatment eligibility between men and women with disease progression: in one study 74% of male versus 37% of female progressors were eligible for therapy in the first year after seroconversion [64]. Setting aside the clinical questions regarding treatment initiation, the viral load gap between men and women also opened significant questions about disease pathogenesis and the determinants of an effective immune response.

The viral load of an individual infected with HIV is determined by a combination of the characteristics of the infecting virus itself and the host immune response, with the most extreme examples seen in patients who spontaneously suppress or control viremia (reviewed in [70–74]). Women are overrepresented in some cohorts of immune controllers of HIV [75, 76] raising questions about whether sex differences in immune responses track with features linked to spontaneous viral suppression. Given the heterogeneous characteristics of HIV controllers, separating the sex-based components may amplify subtle differences that confer immunologic advantages. A sex-based difference in HIV control is consistent with an extensive body of literature documenting distinctions between the sexes in acquisition and progression of viral infections and in the protective efficacy of and adverse responses to vaccinations [51, 52, 77, 78]. However, there is little evidence to define a mechanism for the difference in HIV infection.

From the standpoint of protective genetic factors, it is notable that many genome association studies, including the International H.I.V. controllers study [79], analyze only the autosomal chromosomes. Given the multiple X-encoded miRNAs and immune regulatory genes, this analysis may have omitted factors with relevance to viral control. As there is evidence that sex-based viral load distinctions are also seen in children prior to puberty [80], the role of chromosomal differences merits consideration. Focused exploration of sex-chromosome-linked genetic determinants in HIV control is increasingly feasible as new analytic methods are developed for including the X chromosome in genetic association studies [81, 82]. In addition, the role of steroid hormones in the direct modulation of HIV transcription will be discussed below.

Overall, HIV viral loads tend to be lower in women during early infection, and there is an enrichment of women among spontaneous and post-treatment controllers. This suggests that either hormonal modulation of virus or immune response, or direct sex-linked genetic variation that alters the immune response is contributing to virologic control. Isolating sex as a variable may highlight the protective pathways.

### HIV specific T cell responses

There are limited studies comparing HIV-specific immunity in men and women, and many cohort studies have limited participation of women [83] leaving many questions about how the sex differences in immune response contribute to viral control. One study specifically quantified CD8^+^ T cell responses in both sexes at two time points after seroconversion, demonstrating that these responses were correlated with CD4^+^ T cell counts in women but not in men [84]. Interestingly, the CD8^+^ T cell responses did not differ in magnitude by sex, leading the authors to suggest that the differences observed in viral load between men and women on the population level may not be directly linked to cytotoxic T cell function [84]. Despite the lack of augmented T cell responses in this single study, in non-HIV studies, there was evidence of an amplified T cell activation and proliferation in mucosal T cells from women [85]. Likewise, gene expression analysis in HIV negative populations showed higher induction of inflammatory pathways in cytotoxic T cells from women after *in vitro* re-stimulation; estrogen receptor elements were identified in the promoter regions of many of these differentially expressed genes [86]. Further work is necessary to parse whether there are sex differences in T cell responses relevant to control of plasma viral load.

T cell activation is part of a profile of chronic immune activation associated with HIV viremia and disease progression. There is an inverse relationship between survival and T cell activation in patients with advanced immunodeficiency [87], and CD8^+^ T cell activation in early disease is a strong predictor of immunologic progression [88]. These findings focused attention on the host immune response as a critical determinant of the pathogenesis of AIDS, irrespective of levels of plasma viral load. Again, biological sex is an important modifier; women have a higher level of CD8^+^ T cell activation when controlled for viral load [89] suggesting an inflammatory mechanism for the relatively more rapid disease progression seen in women at lower viral loads. The drivers of heightened T cell activation are likely to include sex differential innate immune responses including production of IFN-α as detailed below.

### Innate immune responses

Innate immune cells prime and promote adaptive responses, and they also mediate direct host protection through recognition and elimination of infected targets. pDCs are the dominant source of type 1 interferon, and pDCs from women produce higher levels of IFN-α when stimulated with HIV-derived Toll-like receptor ligands (TLRs) [89], one potential driver of CD8^+^ T cell activation. As the X chromosome encodes TLRs that directly sense HIV (including TLR7), the sex chromosome complement likely contributes to the amplified production of interferon. In addition, hormones, in particular estrogen, influence pDC IFN-α production in part through direct effects on the expression of interferon regulatory factor 5 (IRF5) [58, 90, 91]. Prior work has also suggested a role for progesterone in the regulation of pDC function. *In vitro* studies showed a potent inhibitory effect of direct coculture with hormone[92] in contrast to an enhanced IFN-α response from pDCs sampled from high progesterone individuals [89]. As these studies highlight, hormone exposure needs to be considered as a net effect of multiple inputs, obligating careful interpretation of *in vivo* studies.

Genetic associations and *in vitro* studies combine to demonstrate a role in HIV pathogenesis for another innate antiviral effector, the natural killer (NK) cell (reviewed in [93, 94]). NK cell function is highly sex-specific with respect to reproduction, where there is clear evidence of evolutionary interactions between NK-cell receptors and HLA ligands [95], and associations with pregnancy loss and intrauterine growth restriction (reviewed in [96, 97]). There is less data to define the role of sex in modulating the function of NK cells at other sites. Age, sex, and menstrual cycle effects on peripheral NK-cell distribution and function have been reported [98, 99], but it is difficult to interpret the relevance of these variations to disease [100, 101]. More focused research is warranted to determine whether sex is associated with relevant differences in NK-cell activity.

Monocytes are another innate cell population critically important to HIV pathogenesis. Monocyte activation has been linked to soluble markers of inflammation that are associated with morbidity and mortality [102] and have been mechanistically linked to coagulation abnormalities that may contribute to cardiovascular events in HIV-infected individuals [103, 104]. Of note, lipid metabolism pathways are linked to monocyte and innate cellular activation in the general population (reviewed in [105]), and lipids are modulated by sex hormones [106]. Although there are limited studies in patients with HIV, in uninfected populations, lower percentages of CD14^+^CD16^++^ monocytes have been reported in healthy women [107], and increased monocyte activation has been reported in women with systemic lupus erythematosus [108]. Lipid metabolism is linked to innate cellular activation (reviewed in [105]) and the sex hormone effects on lipids [106] are a potential mechanism for differences between men and women. Soluble markers of innate immune activation are predictive of HIV disease outcomes in cohort studies, but it is not clear if their performance is accurate in women. The magnitude of changes in biomarkers after initiation of ART has been reported to differ between men and women, but these studies are difficult to interpret. In many cases, there are different levels of pretreatment elevation, and a smaller net change in women may lead to comparable plasma levels. In that context it is difficult to determine whether there is a relevant sex difference in treatment response [109–111].

### Humoral immunity

Sex differences extend to antibody formation, with more robust induction of antibody responses to vaccines, higher rates of autoreactive antibodies, and higher baseline levels of some immunoglobulin subclasses in women (reviewed in [112]). There is evidence for a direct effect of sex hormones, most notably in the proposed role of estrogen in promoting somatic hypermutation and decreasing the stringency of selection against autoreactivity [112]. In contrast, testosterone has been linked to a profile of lipid metabolism that is associated with lower responses to influenza vaccination [113]. Sex differences have been described in many vaccine responses, and the range of ages studied (including prepubertal subjects) suggests that sex hormones are not solely responsible for these differences [40]. In the specific case of HIV, sex differences in the frequency of broadly neutralizing antibody responses (bNabs) to HIV have not been apparent [114], although neutralizing responses are tied to viral load [115], which does have sex specific determinants, as discussed previously.

While a potent pathway for prevention of new infection, neutralization is not the only protective function of antibodies. The modest efficacy seen in the RV144 HIV vaccine trial was associated with non-neutralizing antibody titers [116]. In addition, the antibodies generated by RV144 had more polyfunctional non-neutralizing Fc effector domains when compared to the non-protective VAX003 vaccine trial [117]. Antibody subclass and Fc glycosylation patterns are important determinants of the non-neutralizing functional profiles of antibodies [118]. Ongoing research is exploring the pathways for tuning glycosylation patterns to optimize these antibody functions [119]. Of note, glycan modifications are influenced by age and sex, and levels of estrogen have been linked to a predominance of specific glycoforms [120]. A separate link between the sex hormone milieu and antibody glycan modifications is suggested by the pregnancy-associated changes in rheumatoid arthritis disease activity and the associated patterns of circulating antibody glycosylation [121, 122]. In the tissues of the female reproductive tract, hormones associate with changes in glycosylation machinery [123]. Recent work has directly linked estrogen levels with and without pharmacologic modulation to patterns of glycosylation in bulk IgG [124]; further work is necessary to assess the effects in antigen-specific antibodies. A more detailed understanding of the role of sex and sex hormones in this process may lead to strategies for natural optimization of the antibody glycoprofile in both women and men.

### Microbiome

Inflammation and disease pathogenesis in HIV infection have also been linked to microbial translocation events [125] prompting efforts to shape the gut-associated microbiome to optimize immune parameters [126–128]. Differences in the microbiota of the reproductive tracts with consequences for the efficacy of PrEP have been well described, however, there are also sex differences in the gut microbiota between men and women. Sex hormones favor the predominance of specific microbial communities, and in animal studies they have been linked to the sex-specific susceptibility to autoimmune diseases [129, 130]. Human studies have confirmed the association of sex with specific microbiome characteristics [131–133]. Further studies are needed to define the influence of sex on gut microbial communities and the consequences of a particular microbiome composition for microbial translocation and inflammation.

### Implications

For both the exceptional case of immune control and the more typical phenotype of disease progression, sex differences in HIV infection present several opportunities to further delineate the mechanisms of HIV pathogenesis. A heightened inflammatory response may offer a selective advantage for establishing a state of viral control, but in the more typical case of disease progression, this will confer a risk for amplified pathology and disease progression at lower viral loads. Furthermore, these differences may contribute to inflammatory comorbidities in HIV including cardiovascular disease. Analyses of women with known hormonal status (premenopausal and postmenopausal, exposure to exogenous hormones) will help to define the contribution of hormones, a pathway that can be pharmacologically modified. Likewise, differences in the immune response to infection may also lead to efforts at successful vaccine design.

## HIV CURE

Embedded within the discussion of treated HIV disease is the question of whether sex differences will be relevant to strategies for cure. Again, the role of sex is not fully defined, but the biological distinctions between men and women may offer points of leverage. A successful intervention will either definitively eradicate the latent proviral HIV reservoir (eg, through the “shock and kill” approach) or will establish a state of durable immune control or transcriptional silencing to prevent resurgence of the virus after withdrawal of ART (ie, a “functional cure”) (reviewed in [134–136]). While the case of the Berlin patient [137, 138] stands as a point of optimism for the potential to achieve a durable cure of HIV, subsequent cases have demonstrated the challenges of achieving viral eradication [139–142]. The recently described post-treatment controller (PTC) cohort of individuals with durable suppression of viral replication after withdrawal of ART offers a natural model of the goal of functional cure [143]. Of note, as with immune controllers, an association between female sex and PTC status has been reported, albeit based on small numbers [144]. The field of HIV cure research has advanced rapidly in both the basic discovery phase and through clinical trials, but women are underrepresented in these studies [145]. Their limited representation may lead to missed opportunities to identify sources of biological variability that may have relevance. Importantly, many of the curative interventions proposed target the host immune system and not the virus; this means that there is a greater chance of sex differences in responses, which should be carefully considered while therapies are developed and tested.

To frame the discussion of potential sex differences, we will divide cure research into a few domains. Areas of active investigation include 1) defining the size and location of the reservoir, 2) identifying pathways to latency reversal or more permanent silencing of transcription, and 3) mobilizing the immune response to clear infected cells. We will focus the discussion on the first 2 points here, as sex differences in the priming and direction of immune responses via vaccination and following infection were discussed previously.

### Defining the reservoir

Defining the reservoir will identify the targets and delineate the threshold required to eliminate or silence HIV [146]. Several measures have been proposed, each with their caveats. Measurement of the proviral DNA reservoir of HIV has been shown to be predictive of the time to rebound after interruption of therapy in a clinical trial [147]. The HIV DNA reservoir prior to therapy is correlated with plasma viral load and had clinical prognostic value for untreated disease progression [147, 148]. This DNA reservoir is established early during infection and is relatively stable, although it can be significantly reduced by the initiation of ART [149]. The measure also has important limitations, as *ex vivo* studies have demonstrated that a significant proportion of proviruses measured in HIV DNA assays are defective at the sequence level [150], even when measured during acute infection [151], and are not a measure of the replication competent reservoir. Of note, viral RNA species from defective sequences [152] may also contribute to immune activation and pathogenesis, suggesting an importance of the DNA reservoir irrespective of replication competence. However, it is a challenge that there is limited correlation between measures of HIV DNA, RNA, and outgrowth of viruses *in vitro* [153], and the factors governing the establishment of the DNA reservoir are incompletely defined. Two cross-sectional studies with women participants have suggested that female sex is associated with a lower HIV DNA reservoir in peripheral blood mononuclear cells (PBMCs) [154, 155]. These studies were not specifically designed to test sex as a variable, and approximately 30% of the participants were women. In contrast, data from a prospectively recruited cohort of matched men and women showed no significant differences in HIV DNA reservoir measures in CD4^+^ T- cells [156].

Residual HIV expression measured by HIV RNA expression offers a different index of virus activity. Measurements of both cell-associated unspliced (CA-US) and multiply-spliced (CA-MS) RNA transcripts have been used in a variety of studies to quantify HIV expression, although the precise significance of these types of transcripts is a topic of ongoing discussion (reviewed in [157]). The level of CA-RNA was reported to associate with time to rebound in one cohort undergoing an analytic treatment interruption [158], suggesting that this measure may have utility in assessing the potential for cure. Women have lower levels of CA-MS RNA transcripts and lower levels of plasma HIV RNA as measured by a single copy assay [156]. These results suggest that in this particular cohort in which HIV DNA levels were comparable, there may be important differences in HIV RNA production and residual viremia. Measurement of *in vitro* production of replication-competent virus, which has been proposed as the measure most tied to risk of rebound viremia, has not been systematically compared between men and women.

### HIV RNA expression and latency reversal

Sex steroids may contribute to differences in CA-RNA levels through transcriptional control. The estrogen receptor associates with the HIV-1 long terminal repeat in combination with other transcription factors, and 17β-estradiol has an inhibitory effect on HIV replication [159, 160]. Another study using combinations of estrogen and progesterone suggested that high levels of hormone exposure inhibited and lower levels enhanced HIV transcriptional activity [161], although this study did not isolate transcriptional effects and bystander cytokine secretion may have also played a role. Using a small hairpin RNA knockdown screen, Karn and colleagues identified the estrogen receptor as a major regulator of HIV latency reversal in cell line and primary cell models; latency reversal with a variety of activating stimulants was more efficient when estrogen was blocked [162]. This observation was further validated with ex vivo reactivation of primary human samples. In these studies, exposure to estrogen blunted the induction of HIV RNA production after stimulation of the T cell receptor or exposure to histone deacteylase inhibitors [162]. Notably, the repressive effect of estrogen was most pronounced in cells taken from women, *ex vivo* stimulation of cells from men also showed a blunting of HIV expression by estrogen, but with a smaller effect size [162]. Taken together, the data suggest that HIV transcription is directly affected by sex steroids, offering a novel pathway that can be modulated to enhance efforts at reactivation.

The precise determinants of latency maintenance and the ideal pathway for selective activation of HIV transcription remain open questions (reviewed in [163]). Current efforts at latency reversal have included a significant focus on the histone deacetylase inhibitor class of chromatin remodeling agents (reviewed in [164]). Distinct methylation and transcriptional profiles of immune cells from men and women underline the epigenetic controls of sex differences [165]; these differences may have implications for the efficacy of chromatin-remodeling agents in HIV latency reversal. Other stages between a latent provirus and production of a replication- competent viral particle such as post-transcriptional and post-translational modifications and trafficking may also be targets for functional cure strategies. To date, there are no specific data to suggest sex differences at these stages in the viral life cycle.

### Immunomodulatory therapies

Other agents in preclinical or early clinical trials for HIV cure include immunomodulators including checkpoint inhibitors such as anti-PD1. Sex has recently been identified as a predictor of response to anti-PD1 therapy in the setting of cancer [166]. It is not known whether these differences reflect sex effects on the therapy or on the tumor, but given the differences in immune phenotypes between men and women, responses to checkpoint inhibitor therapy in women in all settings should be carefully monitored. The TLR7 agonists are another class of agents under evaluation for latency reversal; as detailed previously, *TLR7* is encoded on the X chromosome and both gene dosage [57] and hormonal effects [58] lead to sex specificity in function. Women may have a higher likelihood of both adverse effects and positive responses to this type of therapy, and sex-stratified analyses should be explored.

Threading through this discussion is the subtext that women must be included in clinical trials in order to clearly assess sex-specific effects. A challenge to those efforts is the potential for reproductive toxicity of some of the therapies in clinical trials. Policy guidelines from the NIH on the inclusion of women in research [167] and the American College of Gynecology and Obstetrics (ACOG) on the inclusion of pregnant women [168] offer guidance on the inclusion of this more “scientifically complex” population. These factors must be considered together with the specific risks and ethical considerations in HIV-cure research along with ongoing engagement of the community living with HIV. Efforts should be made to include women in trials relevant to cure and to evaluate the efficacy of these agents in a sex-specific manner whenever feasible.

### Implications

Sex differences in reservoir size, location (anatomic and cellular compartments), residual activity, integrity (ie, frequency of defective sequences), and inducibility are all possible based on the known biological differences between men and women. This represents both a challenge and an opportunity. The challenge is to include women in clinical trials while accounting for the inconvenient variance associated with hormone fluctuations, reproductive risks associated with some latency reversal agents, and the difficulty enrolling women in clinical trials. The opportunity lies in the fact that some of these biological differences will point to important mechanistic pathways that determine reservoir size and replication competence. These pathways may include direct sex hormone activity or immune factors such as the type 1 interferons.

Initial findings regarding the effect of estrogen on latency reversal have provided the basis for development of a clinical trial to test the potential for synergistic reactivation of HIV latency with estrogen blockade combined with transcriptional activators [169]. This is only the first example of how a comparative biology approach may highlight novel therapeutic pathways relevant to HIV cure.

## CONCLUSIONS

Women bear a substantial share of the burden of HIV infection worldwide and remain underrep-resented in clinical studies [83] and specifically in trials relevant to cure [145]. At every stage of infection from acquisition, treatment and pathogenesis, and cure, there are sex differences in the response to HIV (Table 1). Disentangling the effects of hormone exposure from genetic determinants is 1 strategy for identifying therapeutic targets. Enrolling women who are premenopausal or postmenopausal, and studying the effects of hormones in transgender individuals may help us isolate the contribution of hormone exposure. Studies with low or unbalanced representation of women may inappropriately attribute a sex-driven difference in inflammatory or virologic characteristics to an intervention such as vaccination or a cure strategy. When enrollment of women is limited, aggregation of data from multiple sources may allow more balanced assessment of sex-specific risks. There is a need for sex-stratified analyses, proportional inclusion of women, and clinical trials to investigate biological pathways that may differ between women and men. This research would offer the promise of not only uncovering pathways relevant to women, but also of clarifying the precise regulatory pathways that may reveal interventions relevant to both men and women.

**Table 1:** 

*Feature of HIV*	*Sex difference*	*Postulated mechanisms*
Acquisition	Enhanced risk of sexual transmission to women	-anatomic differences in mucosal surfaces -hormonal modulation of target cells at mucosal sites -local inflammation in response to sexually transmitted diseases increasing target cells
	Inconsistent effcacy of PrEP in women	-sociobehavioral determinants of adherence -microbiome-mediated reduction of local tenofovir concentrations in the female genital tract
	Vaccine effectiveness	-X chromosome genes and regulatory elements that affect the immune response to vaccines.
Pathogenesis	Lower setpoint viral loads in women during early infection	more effcient/robust type 1 interferon response in women -more effcient adaptive immune response -estrogen effects on transcription
	Enrichment of women in post treatment and spontaneous controllers	
	Enhanced risk of comorbid disease in HIV-infected women	-greater relative risk of comorbidities driven by inflammation/immune activation
Cure	Proviral reservoir comparable but decreased measures of reservoir activity in women	-estrogen control of transcription -differences in quality of proviral reservoir -differences in inducibility or T cell subset
	Curative interventions targeting host immune responses	-sex differences in epigenetic structure may modulate efficacy of chromatin modifiers -sex is a predictor of checkpoint inhibitor performance in cancer -TLR7 agonists may have sex differential effects given baseline sex differences in activity and expression
